# Induced Sporicidal Activity of Chlorhexidine against *Clostridium difficile* Spores under Altered Physical and Chemical Conditions

**DOI:** 10.1371/journal.pone.0123809

**Published:** 2015-04-10

**Authors:** Michelle M. Nerandzic, Curtis J. Donskey

**Affiliations:** 1 Research Service, Cleveland Veterans Affairs Medical Center, Cleveland, Ohio, United States of America; 2 Geriatric Research, Education and Clinical Center, Cleveland Veterans Affairs Medical Center, Cleveland, Ohio, United States of America; Institute Pasteur, FRANCE

## Abstract

**Background:**

Chlorhexidine is a broad-spectrum antimicrobial commonly used to disinfect the skin of patients to reduce the risk of healthcare-associated infections. Because chlorhexidine is not sporicidal, it is not anticipated that it would have an impact on skin contamination with *Clostridium difficile*, the most important cause of healthcare-associated diarrhea. However, although chlorhexidine is not sporicidal as it is used in healthcare settings, it has been reported to kill spores of Bacillus species under altered physical and chemical conditions that disrupt the spore’s protective barriers (e.g., heat, ultrasonication, alcohol, or elevated pH). Here, we tested the hypothesis that similarly altered physical and chemical conditions result in enhanced sporicidal activity of chlorhexidine against *C*. *difficile* spores.

**Principal Findings:**

*C*. *difficile* spores became susceptible to heat killing at 80°C within 15 minutes in the presence of chlorhexidine, as opposed to spores suspended in water which remained viable. The extent to which the spores were reduced was directly proportional to the concentration of chlorhexidine in solution, with no viable spores recovered after 15 minutes of incubation in 0.04%–0.0004% w/v chlorhexidine solutions at 80°C. Reduction of spores exposed to 4% w/v chlorhexidine solutions at moderate temperatures (37°C and 55°C) was enhanced by the presence of 70% ethanol. However, complete elimination of spores was not achieved until 3 hours of incubation at 55°C. Elevating the pH to ≥9.5 significantly enhanced the killing of spores in either aqueous or alcoholic chlorhexidine solutions.

**Conclusions:**

Physical and chemical conditions that alter the protective barriers of *C*. *difficile* spores convey sporicidal activity to chlorhexidine. Further studies are necessary to identify additional agents that may allow chlorhexidine to reach its target within the spore.

## Introduction

Chlorhexidine is a cationic bisbiguanide with activity against Gram-negative and Gram-positive bacteria, yeasts, and enveloped viruses [1–3]. Due to its broad-spectrum antimicrobial activity, chlorhexidine is used in a wide variety of disinfectant, antiseptic and preservative applications [2]. In healthcare settings, chlorhexidine is routinely used to disinfect the skin of patients prior to surgical procedures and catheter insertion to reduce the risk of healthcare-associated infections [4,5]. Furthermore, daily bathing of patients with chlorhexidine gluconate (CHG) has become increasingly prevalent because it has been shown to reduce the incidence of bloodstream infections and acquisition of multidrug-resistant organisms, including methicillin-resistant *Staphylococcus aureus* (MRSA) and vancomycin-resistant enterococci (VRE) [6–10].

*Clostridium difficile* is an anaerobic, spore-forming bacterium that is the most important cause of healthcare-associated diarrhea [11,12]. Patients with *C*. *difficile* infection (CDI) shed spores in stool, resulting in contamination of their skin, clothing, and environmental surfaces [13]. *C*. *difficile* spores on skin are considered a major source of transmission because they can easily be acquired on the hands of healthcare workers [14–16]. In addition, skin contamination could contribute to recurrence of infection if spores are transferred to the hands of CDI patients and ingested. Thus, effective methods to reduce the burden of spores on skin could be helpful to reduce transmission and recurrence. Although showering reduced the burden of *C*. *difficile* spores on CDI patients’ skin to a modest degree through mechanical removal, bed baths using soap and water were ineffective [17]. Because chlorhexidine is not a sporicidal agent as applied in healthcare settings, it is not anticipated that CHG bathing, particularly when applied as a bed bath, would have an impact on the burden of spores on skin or on the incidence of CDI. However, Rupp et al. [18] recently reported that hospital-wide CHG patient bathing was associated with a significant reduction in the incidence of healthcare-associated CDI. The basis for this unexpected finding is unclear since the impact of bathing on the burden of spores on skin was not assessed.

Chlorhexidine is a membrane active compound that disrupts the protective barriers of vegetative organisms by interacting with the negative charges associated with the cell wall and plasma membrane [19,20]. Bacterial spores have several proteinaceous coats that surround and protect the dormant bacterial cell wall and membrane, making the antimicrobial properties of chlorhexidine ineffective against intact dormant spores [21,22]. Nonetheless, by applying physical and chemical conditions that degrade or allow penetration of the protective spore coats, chlorhexidine may reach its target within the spore [23–25]. For example, chlorhexidine does not kill dormant spores under ambient conditions, but as demonstrated in *Bacillus* species, sporicidal activity is observed at elevated temperatures. *Bacillus* spores suspended in 0.01% to 1% CHG solutions were reduced by 5 log_10_ colony forming units (CFU) after 6 minutes of exposure to temperatures of 98–100°C, as compared to a 1 log_10_CFU reduction in the absence of chlorhexidine [23]. In conjunction with sporicidal activity induced at elevated temperatures, several studies have reported that the presence of alcohol, elevated pH, and ultra-sonication enhance the sporicidal character of chlorhexidine at sub-lethal temperatures ranging from 37–55°C [23–25]. As a consequence, chlorhexidine has been recommended for cold liquid chemo-sterilization of thermolabile equipment and materials at moderate temperatures with the addition of an enhancing agent such as ultra-sonication, alcohol, or elevated pH [23].

Previous studies have not examined the impact of altered physical and chemical conditions on activity of chlorhexidine against *C*. *difficile* spores. Therefore, we tested the hypothesis that similarly altered physical and chemical conditions result in enhanced sporicidal activity of chlorhexidine against *C*. *difficile* spores. Initially, we examined the effects of chlorhexidine gluconate (CHG) and chlorhexidine free base (CHX) on *C*. *difficile* spores at 80°C. Next, we determined the effects of ethanol and isopropanol on sub-lethal heat treatments in the presence of CHG. Finally, we assessed the impact of pH on the sporicidal character of chlorhexidine at elevated temperatures.

## Materials and Methods

### *Clostridium difficile* Strains

Two strains cultured from patients with CDI at the Cleveland VA Medical Center were studied. VA 17 is an epidemic (cdtB+) restriction endonuclease analysis (REA) BI strain and VA 11 is a non-epidemic (cdtB-) REA J strain. Both isolates are toxigenic (tcdA+, tcdB+) strains. The Institutional Review Board of the Cleveland VA Medical Center approved the study protocol for collection of all patient isolates. Informed consent was not obtained because the isolates were cultured from clinical samples with no collection of patient identifiers or interaction with subjects.

### Preparation of *Clostridium difficile* Spores

*C*. *difficile* spores were prepared as previously described [26]. In brief, pre-reduced brain-heart infusion plates were spread with 100 μl of a 24-hour suspension of a culture of *C*. *difficile* and incubated for one week in a Whitley MG1000 anaerobic workstation (Microbiology International, Frederick, MD). Spores were harvested from 10 plates using sterile swabs and 8 mL of ice-cold, sterile, distilled water. Spores were washed five times by centrifuging at 15,000 x g for 5 min and re-suspending in distilled water. After washing, the spores were collected in 1 mL of 20% (w/v) HistoDenz and layered onto 20 mL of 50% (w/v) HistoDenz solution. The gradient was centrifuged at 15,000 x g for 15 min and the spore pellet was carefully collected from the bottom of the tube and washed with distilled water three times. Spores were stored at 4°C in sterile distilled water until use. Prior to testing, spore preps were confirmed by phase contrast microscopy and malachite green staining to be >99% dormant, bright-phase spores.

### Heat Susceptibility of *Clostridium difficile* Spores Exposed to Chlorhexidine

Dormant *C*. *difficile* spores remain 100% viable when incubated at 80°C for up to 15 minutes [27]. Initial experiments were performed to assess the viability of *C*. *difficile* spores in the presence of chlorhexidine free base (Sigma-Aldrich, St. Louis, MO) or chlorhexidine gluconate (Sigma-Aldrich, St. Louis, MO) at 80°C. Solutions of chlorhexidine free base (CHX) and chlorhexidine gluconate (CHG) were prepared in sterile deionized water at concentrations of 0.4 (0.04% w/v), 0.04 (0.004% w/v), and 0.004 (0.0004% w/v) mg/mL. Ten microliter aliquots of *C*. *difficile* spores (5 log_10_CFU VA11 or VA17) were suspended in 1 mL of CHX, CHG, or sterile deionized water (positive control). Spore suspensions were incubated in an 80°C water bath for 5, 10, or 15 minutes. Vegetative *C*. *difficile* is acutely sensitive to chlorhexidine; therefore, to ensure that organisms were not inhibited from growing due solely to the presence of chlorhexidine, spores were incubated in water, CHX, and CHG preparations at room temperature (~22°C) to serve as verification of unrestricted outgrowth.

To quantify viable organisms, aliquots of the spore suspensions were neutralized 1:1 in Dey-Engley neutralizer (Becton Dickinson, Cockeysville, MD), then serially diluted and drop-plated onto pre-reduced cycloserine-cefoxitin-brucella agar containing 0.1% taurocholic acid and lysozyme 5 mg/L (CDBA) in a Whitley Workstation MG1000 anaerobic chamber (Microbiology International, Frederick, MD). For samples that yielded below the limit of detection following the serial dilution plating method, experiments were repeated and 1 mL of the neutralized sample was spread onto CDBA to detect low levels of *C*. *difficile*. Recovery of zero colony forming units after three spread plate trials was considered 100% reduction. To determine if carry-over of chlorhexidine was effectively neutralized and not affecting recovery of viable vegetative organisms, the American Society for Testing and Materials “Standard Test Methods for Evaluation of Inactivators of Antimicrobial Agents” was performed for the concentration of chlorhexidine in spread plate samples [28]. Following 48 hours of incubation at 37°C, log_10_CFU reductions were calculated by comparing the log_10_CFU recovered from chlorhexidine solutions to untreated controls (spores suspended in water). All experiments were repeated four times.

### The Effect of Alcohol on Heat Killing of *Clostridium difficile* Spores Exposed to Chlorhexidine

It has been previously demonstrated that alcohol enhances heat killing of *Bacillus* spores exposed to chlorhexidine [23–25]. To determine if heat killing of *C*. *difficile* spores is similarly enhanced by alcoholic chlorhexidine solutions, solutions of 4% (40mg/mL) CHG were prepared in sterile deionized water (aqueous CHG) or 70% ethanol (alcoholic CHG). Ten microliter aliquots of *C*. *difficile* spores (6 log_10_ CFU, VA17) were suspended in 1 mL of aqueous CHG, alcoholic CHG, or sterile deionized water (positive control). Spore suspensions were incubated at room temperature (20°C), 37°C, or 55°C in a water bath for 0, 1, 2, and 3 hours. At each time point, aliquots of the spore suspensions were neutralized 1:1 in Dey-Engley neutralizer (Becton Dickinson, Cockeysville, MD) and viable organisms were enumerated as described above in *Heat Susceptibility of C*. *difficile Spores Exposed to Chlorhexidine*. For samples that yielded below the limit of detection following the serial dilution plating method, experiments were repeated and 1 mL of the neutralized sample was spread onto CDBA to detect low levels of *C*. *difficile*. Recovery of zero colony forming units after three spread plate trials was considered 100% reduction. To determine if carry-over of chlorhexidine was effectively neutralized and not affecting recovery of viable vegetative organisms, the American Society for Testing and Materials “Standard Test Methods for Evaluation of Inactivators of Antimicrobial Agents” was performed for the concentration of chlorhexidine in spread plate samples [28]. Log_10_CFU reductions were calculated by comparing the log_10_CFU recovered from chlorhexidine solutions to untreated controls (spores suspended in water). All experiments were repeated three times.

Additionally, ethanol and isopropanol were comparatively assessed to determine whether the form of alcohol had an impact on heat killing of spores exposed to chlorhexidine. Solutions of CHG (4% and 0.04%) were prepared in water, 70% isopropanol, or 70% ethanol. Ten microliter aliquots of *C*. *difficile* spores (6 log_10_CFU, VA17) were suspended in 1 mL of aqueous CHG, alcoholic CHG (ethanol or isopropanol), or sterile deionized water (positive control). Spore suspensions were incubated at 55°C in a water bath for 0, 1, and 3 hours. At each time point, aliquots of the spore suspensions were neutralized 1:1 in Dey-Engley neutralizer and viable organisms were enumerated as described above in *Heat Susceptibility of C*. *difficile Spores Exposed to Chlorhexidine*. Log_10_CFU reductions were calculated by comparing the log_10_CFU recovered from chlorhexidine solutions to untreated controls (spores suspended in water). All experiments were repeated three times.

### The Impact of pH on Heat Killing of *Clostridium difficile* Spores Exposed to Chlorhexidine

To determine the impact of pH on heat killing of spores exposed to chlorhexidine, 0.04% (0.4 mg/mL) solutions of CHG prepared in water, 70% isopropanol, or 70% ethanol were altered with either hydrochloric acid or sodium hydroxide to a final pH of 4.0, 9.5, or 11.5. Ten microliter aliquots of *C*. *difficile* spores (6 log_10_ CFU VA17) were suspended in 1 mL of pH altered aqueous CHG, alcoholic CHG (ethanol or isopropanol), or sterile deionized water (positive control). Spore suspensions were incubated at 55°C in a water bath for 0, 1, and 3 hours. At each time point, aliquots of the spore suspensions were neutralized 1:1 in Dey-Engley neutralizer (Becton Dickinson, Cockeysville, MD) and viable organisms were enumerated as described above in *Heat Susceptibility of C*. *difficile Spores Exposed to Chlorhexidine*. For samples that yielded below the limit of detection following the serial dilution plating method, experiments were repeated and 1 mL of the neutralized sample was spread onto CDBA to detect low levels of *C*. *difficile*. Recovery of zero colony forming units after three spread plate trials was considered 100% reduction. To determine if carry-over of chlorhexidine was effectively neutralized and not affecting recovery of viable vegetative organisms, the American Society for Testing and Materials “Standard Test Methods for Evaluation of Inactivators of Antimicrobial Agents” was performed for the concentration of chlorhexidine in spread plate samples [28]. Log_10_CFU reductions were calculated by comparing the log_10_CFU recovered from chlorhexidine solutions to untreated controls (spores suspended in water). All experiments were repeated three times.

### Data Analysis

Data were analyzed using STATA 9.0 (StataCorp, College Station, TX). Continuous data were analyzed using unpaired *t* tests. The means of the data from experiments conducted are presented. Error bars indicate standard error.

## Results

### Heat Susceptibility of *Clostridium difficile* Spores Exposed to Chlorhexidine

**Fig 1:**
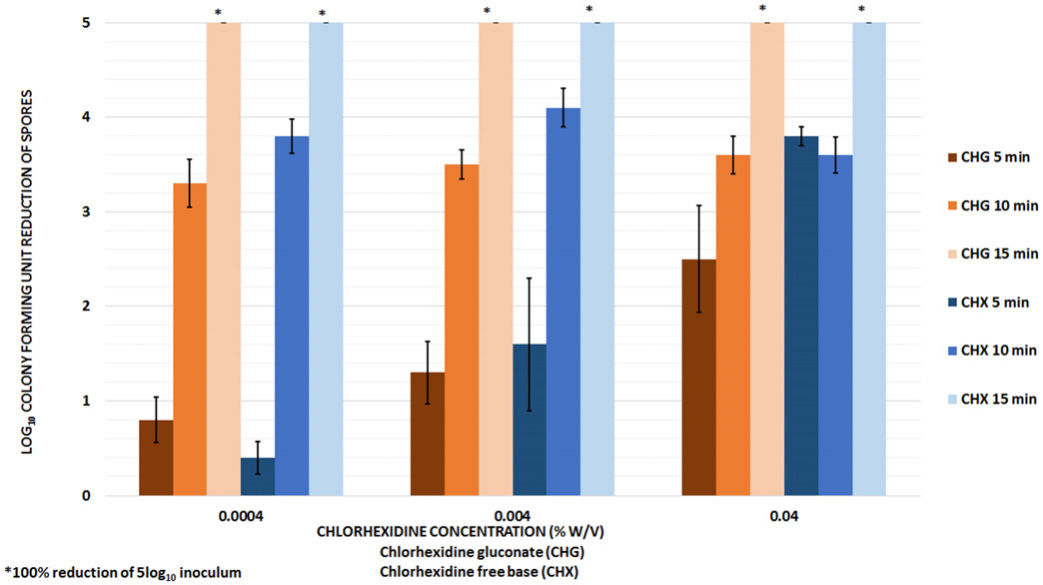
Heat killing of *Clostridium difficile* spores exposed to chlorhexidine gluconate (CHG) and chlorhexidine free base (CHX) solutions. The mean log_10_colony-forming unit (CFU) reduction of *C*. *difficile* spores exposed to CHG and CHX solutions at 80°C. After 5 minutes of exposure to CHG or CHX, heat killing increased as the concentration of chlorhexidine was increased. However, after 10 or 15 minutes of exposure to chlorhexidine at 80°C, similar reductions were achieved at each concentration. The means of the data from four experiments conducted are presented. Error bars indicate standard error.

Fig 1 shows the mean log_10_CFU reduction of *C*. *difficile* spores exposed to CHG and CHX solutions at 80°C. There was no significant difference in the reductions achieved by the two strains of *C*. *difficile* spores assessed (VA11 and VA17); therefore, data for both strains were pooled for analysis (*P* >0.01 for each comparison). Spores suspended in sterile water were not killed by heating to 80°C for 15 minutes. Additionally, neutralization was shown to be effective for all concentrations of chlorhexidine assessed, therefore, killing of spores was not an artifact of inhibition of growing vegetative organisms due to carry-over of chlorhexidine onto culture media. There was no significant difference in the reductions achieved by equivalent concentrations of CHG or CHX solutions (*P* >0.01 for each concentration compared); therefore, in subsequent experiments CHG solutions were used because it is readily soluble in aqueous and alcoholic solvents. After 5 minutes of exposure to chlorhexidine (CHG or CHX) at 80°C, the killing effects of heat and chlorhexidine increased as the concentration of chlorhexidine was increased (<1log_10_CFU reduction for 0.004 mg/mL, >1 log_10_CFU reduction for 0.04 mg/mL, and >2 log_10_CFU reduction for 0.4 mg/mL). However, after 10 or 15 minutes of exposure to chlorhexidine at 80°C, similar reductions were achieved at each concentration (>3 log_10_CFU reduction after 10 minutes and ≥5 log_10_CFU reduction after 15 minutes). Complete elimination of spores was observed after 15 minutes of exposure to CHG or CHX solutions at 80°C.

### The Effect of Alcohol on Heat Killing of *Clostridium difficile* Spores Exposed to Chlorhexidine

**Fig 2:**
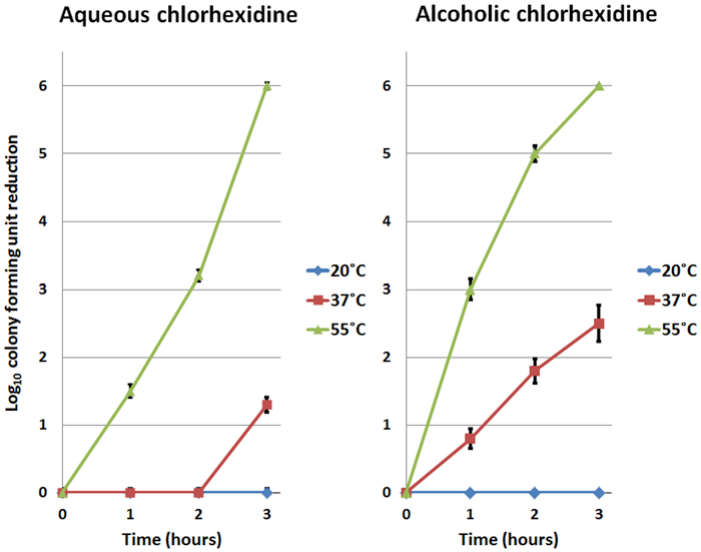
Enhancement of heat killing of *Clostridium difficile* spores exposed to alcoholic chlorhexidine gluconate (CHG) solutions. The mean log_10_colony-forming unit (CFU) reduction of *C*. *difficile* spores exposed to 4% w/v CHG solution prepared in water or 70% ethanol. No killing of spores was observed in aqueous or alcoholic chlorhexidine solutions at 20°C. At 37°C, the presence of alcohol reduced the incubation time required to achieve an ~1 log_10_CFU reduction from 3 hours to 1 hour. At 55°C, alcohol boosted spore reductions from 1.5 log_10_CFU (aqueous) to 3log_10_CFU (alcoholic), and 3log_10_CFU (aqueous) to 5log_10_CFU (alcoholic) after 1 and 2 hours respectively. The means of the data from experiments conducted in triplicate are presented. Error bars indicate standard error.

Fig 2 demonstrates that heat killing of *C*. *difficile* spores exposed to 4% (40 mg/mL) chlorhexidine was enhanced by the presence of 70% ethanol. No killing of spores was observed in aqueous or alcoholic chlorhexidine solutions at room temperature (20°C). At 37°C, spores exposed to aqueous chlorhexidine solution were reduced by ~1log_10_CFU after 3 hours of incubation. Alcohol enhanced spore killing at 37°C, reducing the incubation time from 3 hours to 1 hour to achieve ~1log_10_CFU reduction. Moreover, alcohol augmented the killing of spores after 3 hours of incubation at 37°C, increasing the reduction to >2log_10_CFU. At 55°C, alcohol boosted spore killing after 1 and 2 hours of incubation from 1.5 log_10_CFU (aqueous) to 3log_10_CFU (alcoholic), and 3log_10_CFU (aqueous) to 5log_10_CFU (alcoholic), respectively. Spores were 100% eliminated after 3 hours of exposure to either aqueous or alcoholic chlorhexidine solution at 55°C.

**Fig 3:**
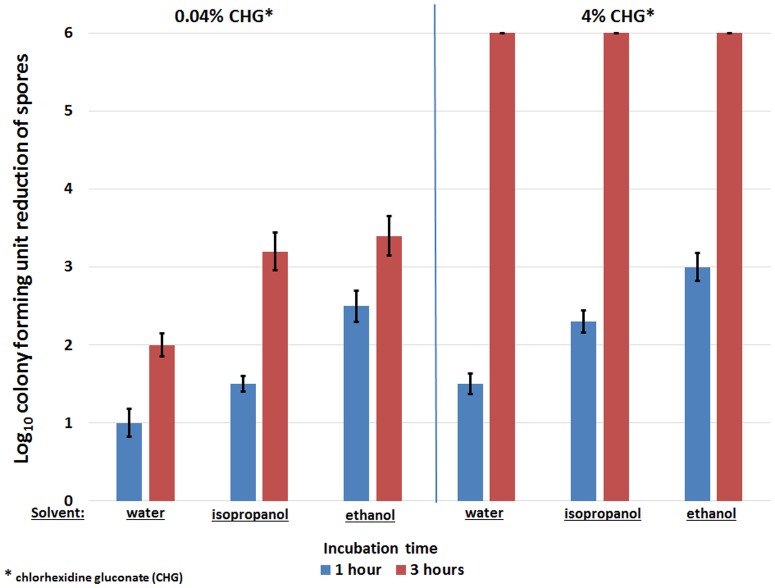
Comparison of heat killing of *Clostridium difficile* spores in chlorhexidine gluconate (CHG) solutions prepared with isopropanol or ethanol. The mean log_10_colony-forming unit (CFU) reductions of *C*. *difficile* spores achieved after 1 or 3 hours of exposure to 0.04% or 4% w/v CHG prepared in water, 70% isopropanol, or 70% ethanol at 55°C. CHG solutions prepared with ethanol significantly enhanced heat killing of spores after 1 hour of incubation compared to CHG solutions prepared in either isopropanol or water (*P* <0.01 compared to isopropanol; *P* <0.001 compared to water). After 3 hours of incubation in 0.04% w/v CHG, both isopropanol and ethanol enhanced reduction of spores compared to aqueous CHG solution; however, at increased CHG concentrations (4% w/v), spores were completely eliminated by both aqueous and alcoholic preparations. The means of the data from experiments conducted in triplicate are presented. Error bars indicate standard error.

Fig 3 shows the difference in the mean log_10_CFU reductions of *C*. *difficile* spores achieved after 1 or 3 hours of exposure to CHG prepared in water, 70% isopropanol, or 70% ethanol at 55°C. CHG solutions prepared with ethanol significantly enhanced heat killing of spores after 1 hour of incubation compared to CHG solutions prepared in either isopropanol or water (*P*<0.01 compared to isopropanol; *P*<0.001 compared to water). When exposed to lower concentrations of CHG (0.04%), the presence of alcohol (isopropanol or ethanol) significantly enhanced reduction of spores after 1 or 3 hours of incubation compared to 0.04% CHG prepared in water. However, when the concentration of CHG was increased to 4%, the presence of alcohol (isopropanol and ethanol) only significantly enhanced heat killing of spores after 1 hour of incubation, because 100% reduction of spores was achieved by both aqueous and alcoholic CHG solutions after 3 hours of incubation.

### The Impact of pH on Heat Killing of *Clostridium difficile* Spores Exposed to Chlorhexidine

**Fig 4:**
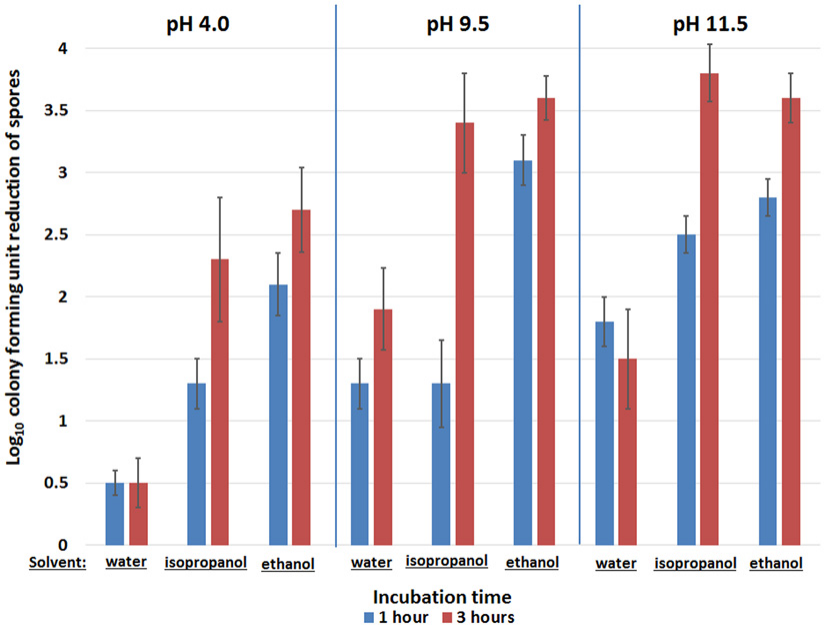
The Impact of pH on heat killing of *Clostridium difficile* spores exposed to chlorhexidine gluconate (CHG). The mean log_10_colony-forming unit (CFU) reductions of *C*. *difficile* spores achieved after 1 or 3 hours of incubation in pH altered aqueous and alcoholic CHG solutions (0.04% w/v). Elevating the pH to ≥9.5 significantly enhanced the killing of spores in either aqueous or alcoholic CHG solutions (*P* <0.001 for each comparison to pH 4.0). In aqueous or alcoholic CHG solutions, increasing the pH to ≥9.5 enhanced heat killing of spores by ≥1log_10_CFU after 3 hours of incubation. The means of the data from experiments conducted in triplicate are presented. Error bars indicate standard error.

Fig 4 demonstrates the effect of pH on heat killing of spores (55°C) exposed to aqueous and alcoholic CHG solutions (0.04% chlorhexidine gluconate). Elevating the pH to ≥9.5 significantly enhanced the killing of spores in either aqueous or alcoholic CHG solutions after 1 and 3 hours of incubation (*P*<0.001 for each comparison to pH 4.0). In aqueous CHG solutions, increasing the pH to ≥9.5 enhanced heat killing of spores by ≥1log_10_CFU after 1 or 3 hours of incubation. Similarly, after 3 hours of incubation, increasing the pH to ≥9.5 enhanced heat killing of spores by ≥1log_10_CFU in CHG solutions prepared in isopropanol or ethanol.

## Discussion

We found that in the presence of chlorhexidine (CHG or CHX), *C*. *difficile* spores became susceptible to heat killing at 80°C within 15 minutes, as opposed to spores suspended in water, which remained viable after 15 minutes of incubation at 80°C. The extent to which the spores were reduced was directly proportional to the concentration of chlorhexidine in solution. No viable spores were recovered after 15 minutes of incubation in 0.04%- 0.0004% w/v chlorhexidine solutions at 80°C. Reduction of spores exposed to 4% w/v chlorhexidine solutions at moderate temperatures (37°C and 55°C) was enhanced by the presence of 70% ethanol, but complete elimination of spores was not achieved until 3 hours of incubation at 55°C. Ethanol was superior to isopropanol for enhancement of heat killing at 55°C after 1 hour of incubation, but after 3 hours of incubation isopropanol and ethanol provided equivalent enhancement of heat killing. Elevating the pH to ≥9.5 significantly enhanced the killing of spores in either aqueous or alcoholic chlorhexidine solutions. These data suggest that the altered physical and chemical conditions that result in enhanced sporicidal activity of chlorhexidine against *Bacillus* spp. spores result in similar enhancement of sporicidal activity against *C*. *difficile* spores.

Our findings have several important implications. First, it is impractical to imply that the sporicidal effects of chlorhexidine, alcohol, and high temperatures (or long exposures to more moderate temperatures) would be a promising approach for disinfection of *C*. *difficile* spores from skin or environmental surfaces. However, we can postulate that more benign physical or chemical agents that cause similar denaturation of the spore’s coat might provide a means to enhance the sporicidal activity of chlorhexidine. Future studies may provide insight into alternative means to allow chlorhexidine to reach its target within the dormant spore. Second, as previously demonstrated, the sporicidal activity of chlorhexidine was increased under basic conditions (pH ≥9.5). One potential explanation for this observation is that a basic environment may serve as an additional form of denaturation. It is well documented that base is an effective protein denaturant [29]. Alternatively, under basic conditions chlorhexidine is largely non-ionized [25]. Non-ionized forms of molecules have been shown to more readily permeate the spore’s protective coats [22, 25]. Finally, our findings suggest some potential mechanisms by which CHG bathing as currently practiced could reduce the burden of spores on skin. Previous studies have shown that chlorhexidine has a persistent effect for up to 24 hours after application to the skin [30, 31]. We can postulate that the elevated temperature of skin in combination with the persistent effect of high concentrations of chlorhexidine may reduce spores over extended periods of time. Future studies are necessary to determine the impact of CHG bathing on levels of spores on skin of CDI patients.

Our study has some limitations. First, there is ambiguity in the literature regarding the effect of chlorhexidine on the germination and outgrowth of spores [5, 32]. In the present study we did not determine whether the combination of chlorhexidine and altered physical and chemical conditions induced or inhibited spore germination. Further studies are necessary to ascertain whether germination was stimulated or halted in effected spores. However, spores were neutralized and exposed to rich nutrient media containing specific *C*. *difficile* germinants post treatment. Consequently, the killing effects observed were permanent even under germination stimulation conditions. Additionally, uninhibited outgrowth was confirmed by recovery of viable organisms after exposure to each individual physical or chemical condition assessed (i.e. chlorhexidine alone, heat alone, etc.). Second, the effect of organic load on the efficacy of chlorhexidine’s induced sporicidal activity was not assessed. However, previous studies on *Bacillus* spores showed that organic load did not reduce the killing efficacy of chlorhexidine under altered chemical and physical conditions [23]. Lastly, elevated temperatures were required to actuate chlorhexidine’s sporicidal character, with or without the addition of a secondary enhancement condition (i.e. the presence of alcohol or elevated pH). Further research is necessary to elucidate the changes that heat imparts on the dormant spore which create an opportunistic environment for chlorhexidine’s activity.

## Supporting Information

S1 Fig Heat killing of *Clostridium difficile* spores exposed to chlorhexidine gluconate (CHG) and chlorhexidine free base (CHX) solutions. Raw data and statistical analysis for Fig 1.
(XLSX)

S2 Fig Enhancement of heat killing of *Clostridium difficile* spores exposed to alcoholic chlorhexidine gluconate (CHG) solutions. Raw data and statistical analysis for Fig 2.
(XLSX)

S3 Fig Comparison of heat killing of *Clostridium difficile* spores in chlorhexidine gluconate (CHG) solutions prepared with isopropanol or ethanol. Raw data and statistical analysis for Fig 3.
(XLSX)

S4 Fig The Impact of pH on heat killing of *Clostridium difficile* spores exposed to chlorhexidine gluconate (CHG). Raw data and statistical analysis for Fig 4.
(XLSX)
